# Laparoscopic Ladd’s Procedure for Adult Intestinal Malrotation With Volvulus: A Case Report and Literature Review

**DOI:** 10.7759/cureus.95371

**Published:** 2025-10-25

**Authors:** Auréline Cousinne, Elie Chelala

**Affiliations:** 1 Surgery, Université Libre de Bruxelles, Brussels, BEL; 2 Gastrointestinal Surgery, Centre Hospitalier Interregional Edith Cavell, Brussels, BEL

**Keywords:** adult intestinal malrotation, incomplete intestinal rotation, intestinal occlusion, jejunal volvulus, ladd's band, laparoscopic ladd’s procedure

## Abstract

Intestinal malrotation is a congenital anomaly of the gastrointestinal tract, most often diagnosed in childhood. It results from abnormal rotation during embryonic development. It may present with acute abdomen, volvulus, or intestinal obstruction. In adults, the condition is rare and typically presents with nonspecific symptoms, making diagnosis challenging and often delayed. We report the case of a 29-year-old woman who presented to the emergency department with abdominal pain and vomiting. Abdominal CT scan revealed intestinal malrotation complicated by volvulus. The patient underwent a successful laparoscopic Ladd’s procedure. A brief review of the literature is provided to highlight this uncommon adult presentation.

## Introduction

Intestinal malrotation is a congenital anomaly caused by incomplete or abnormal 270° counterclockwise rotation of the midgut during embryonic development [1-7]. Clinical manifestations often occur during the neonatal period or the first year of life [2,3,6,8,9]. This affects one in 500 births [1,2]. Adult cases are uncommon, and their incidence is estimated to be less than 0.5% [2,3,6,10]. Some patients remain asymptomatic and are diagnosed incidentally [3,6]. Adults more often present with subtle, variable, and nonspecific symptoms [3,5]. The rarity of the disease and its nonspecific clinical presentation in adulthood contribute to diagnostic challenges and delayed treatment [1,3,5,9]. Treatment involves Ladd's procedure, performed by laparoscopy or laparotomy depending on the severity of the distension and distress of the small intestine [11].

Given its unusual presentation beyond childhood and the therapeutic implications, especially with the increasing role of laparoscopy, reporting such cases remains clinically relevant.

## Case presentation

A 29-year-old woman presented to the emergency department of our tertiary hospital with abdominal pain that had begun the previous day. Initially intermittent, the pain became continuous, localized in the epigastric region to the right hypochondrium, and was associated with vomiting and loose stools. No other alarming symptoms were reported. It was the first time she had shown symptoms of this kind.

On examination, the patient was afebrile and hemodynamically stable. The abdomen was soft and compressible, with tenderness in the epigastric region to the right hypochondrium. Intestinal peristalsis was preserved, and there were no signs of peritonitis.

Routine blood tests and an abdominal CT scan were carried out on the day of his admission. The results, including hematological tests, coagulation profile, renal and liver function, serum electrolytes, C-reactive protein (CRP), and beta-HCG levels, were all within normal limits (Table 1).

**Table 1:** Blood test results

Parameter	Results	Reference range	Unit
Hemoglobin (Hb)	11.7	11.7-15.5	g/dL
White Blood Cell Count (WBC)	6.4	4.5-11	x10³/mm³
Platelet Count (PLT)	267	150-400	x10³/mm³
Activated Partial Thromboplastin Time (aPTT)	28	25.1-37	seconds
Prothrombin Time (PT)	81	>70	%
Sodium (Na+)	141	136-145	mmol/L
Potassium (K+)	3.8	3.5-5.1	mmol/L
Chloride (Cl-)	105	98-107	mmol/L
Urea	15	15-40	mg/dl
Creatinine	0.74	0.50-0.90	mg/dL
Estimated GFR	93	>60	mL/min/1.73m³
Total Bilirubin	0.7	<1.2	mg/dL
Direct Bilirubin	0.2	<0.3	mg/dL
Aspartate Aminotransferase (AST)	15	<32	U/L
Alanine Aminotransferase (ALT)	5	<33	U/L
Alkaline Phosphatase (ALP)	70	35 - 104	U/L
Gamma-Glutamyl Transferase (GGT)	9	<36	U/L
Lipase	31	<60	U/L
CRP	<0.6	<5	mg/L
Beta HCG	<1	<5	U/L

**Figure 1:**
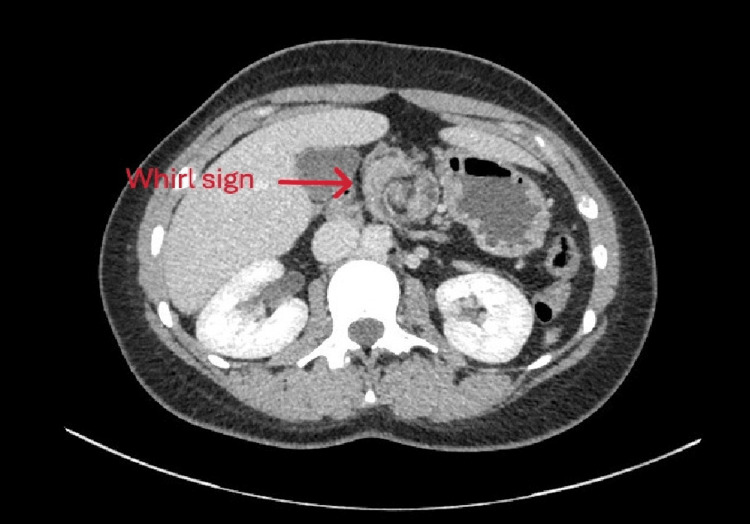
Abdominal CT scan showing a whirl sign, suggesting a volvulus

**Figure 2:**
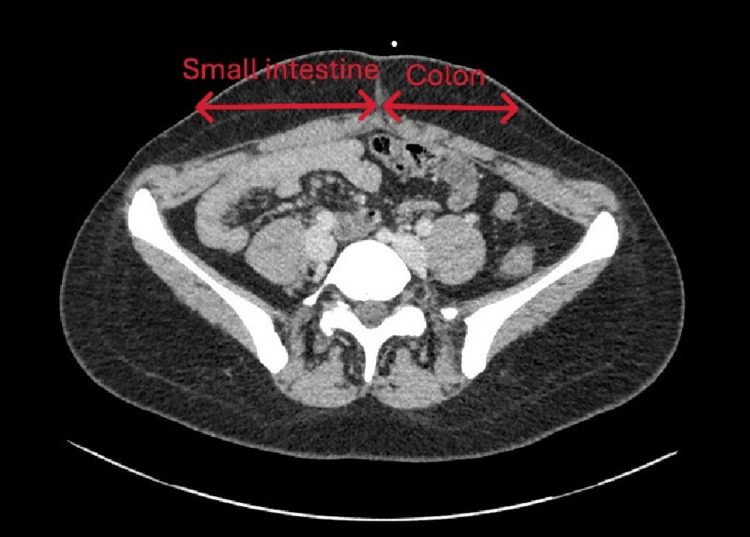
Abdominal CT scan showing the small intestine located on the right side of the abdomen

A contrast-enhanced abdominal CT scan revealed a duodenal twist with small bowel volvulus and abnormal location of the small intestine in the right hemi-abdomen, consistent with intestinal malrotation, but without *signs* of ischemia (Figures 1-2). Gastroscopy showed extrinsic compression of the third portion of the duodenum without stenosis.

**Figure 3:**
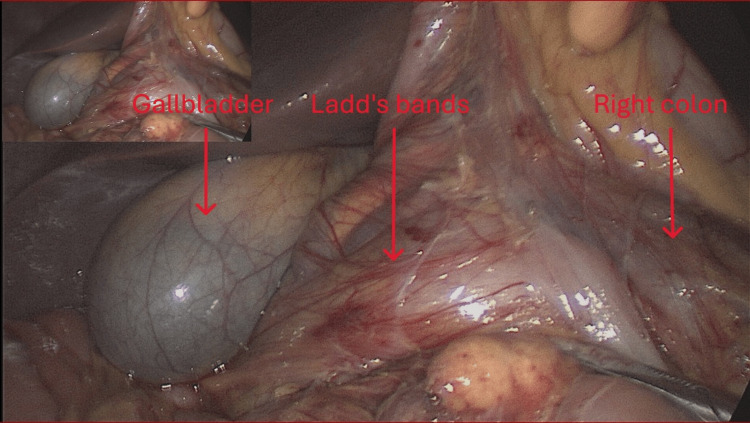
Picture of the procedure showing Ladd’s bands

**Figure 4:**
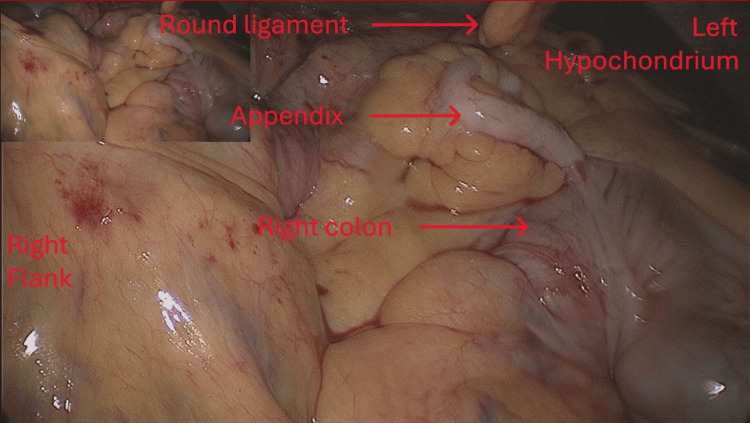
Picture of the procedure showing the appendix on the left side of the abdomen

Given these findings, a diagnostic laparoscopy was performed the day after his admission, using four trocars (one 11 mm trocar at the umbilicus and three 5 mm trocars in the right hypochondrium, left hypochondrium, and epigastrium). The patient was placed supine, anti-Trendelenburg position with legs apart; the surgeon stood between the legs with the assistant on the patient’s left. Exploration revealed partial intestinal malrotation with Ladd’s bands compressing the right colon and duodenum, and a volvulated jejunal loop without signs of ischemia. All Ladd’s bands were divided using a vessel sealing device, and the duodenal adhesions were released. The small intestine was completely untwisted, and the mesentery was positioned in the right hemi-abdomen, with the right colon shifted to the left. The peritoneum between the mesocolon and mesentery was closed with non-absorbable sutures. An appendectomy was also performed to avoid any future confusion and diagnostic errors in cases of acute appendicitis, in accordance with Ladd's procedure (Figures 3-4).

Postoperative recovery was uneventful. The patient resumed oral intake on postoperative day one, tolerated feeding well, and regained normal bowel function by day two. She was discharged home on the second postoperative day with only oral analgesics. Follow-up at 10 days and one month confirmed complete resolution of symptoms, with no abdominal pain and normal bowel transit.

## Discussion

During embryonic development, between the 5th and 10th week, the midgut herniates outside the abdominal cavity before re-entering, rotating 270° counterclockwise around the superior mesenteric artery (SMA) and fixing to the posterior abdominal wall [3,5-7].

Intestinal malrotation results from incomplete or abnormal rotation during embryonic development [1-7]. This leads to a spectrum of intestinal rotation abnormalities, as well as abnormal mesenteric attachment [3,7], malposition of the duodenojejunal junction, Ladd’s bands, and narrow mesenteric insertion [1,3,4,6]. Ladd’s bands are fibrous bands of peritoneum that attach the duodenum and cecum to the posterior wall of the abdomen in the right hypochondrium [3,5,6,11]. These variants predispose to duodenal obstructions and small bowel volvulus [2-5,11].

Most cases are diagnosed in the neonatal period or early infancy [2,3,6,8,9], typically with bilious vomiting, abdominal distension, and acute obstruction [5,9]. Adult presentation is rare and usually nonspecific, with variable symptoms [3,5]. The discovery is sometimes incidental in asymptomatic patients during an abdominal CT scan for another condition [3,6]. Adult patients present with two forms of clinical presentation: chronic or acute [3]. The chronic form is the most common and is characterized by intermittent abdominal pain, vomiting, weight loss, malabsorption, chronic diarrhea, or constipation due to mechanical compression of Ladd’s bands or chronic lymphatic or venous obstruction on a volvulus [2,3,5-8,10,12]. The acute form is characterized by complete cessation of transit and vomiting following intestinal obstruction or volvulus, which can eventually progress to a catastrophic picture of intestinal necrosis with intestinal perforation and peritonitis [2,3,6,8,10,12,13]. The obstruction may also be the result of an internal hernia on Ladd’s bands, which is often underdiagnosed [7].

Due to its rarity and nonspecific symptoms, diagnosis is often difficult or delayed, associated with increased morbidity [1,3,5,9]. A CT scan is the diagnostic modality of choice in adults [3,5,10]. It can demonstrate abnormal bowel positioning, SMA-SMV inversion, absence of pancreatic uncinate process, or the classic “whirlpool sign” of volvulus [3,7]. Doppler ultrasound may also detect vascular inversion or the whirlpool sign [3]. In pediatric patients, an upper gastrointestinal contrast study remains the gold standard, with the characteristic sign being duodenojejunal flexion located to the right of the spine [3,7].

Surgical treatment is indicated in symptomatic patients, with the Ladd procedure being the standard [8-12]. William E Ladd was the first to describe this treatment for malrotation in children in 1936 [3,5,11]. This technique is also performed in adults for all forms of intestinal malrotation [11]. The operation involves counterclockwise detorsion of the bowel [3,5,11,13], division of Ladd’s bands to release dudenal compression [3,5,11,13], broadening of the mesenteric base, appendectomy, and repositioning of the small intestine to the right and the colon to the left [5,11,13]. In our case, mesenteric suturing was performed, but mesenteric fixation is optional [11]. The procedure can be performed via laparotomy or laparoscopy [14]. Laparoscopy offers advantages in terms of recovery, post-operative pain, and aesthetics due to its minimally invasive nature. However, this technique can only be used if the volvulus is reduced or incomplete; otherwise, a laparotomy must be considered [11].

Postoperative outcomes generally depend on the severity of the presentation [12]. Patients with acute volvulus have higher morbidity and mortality [12], whereas elective cases usually recover uneventfully. Persistent gastrointestinal complaints such as abdominal pain, altered bowel habits, or reflux may still occur in some patients despite surgery [9]. Recurrence of volvulus is rare but possible [12].

## Conclusions

Intestinal malrotation is rare in adults and typically presents with nonspecific symptoms, making diagnosis challenging and often delayed. An abdominal CT scan plays a pivotal role in establishing the diagnosis. Early recognition and prompt surgical management are essential to prevent life-threatening complications such as ischemia or perforation. Ladd's procedure remains the gold standard for treatment, and laparoscopy offers a safe and effective approach in selected patients.
